# Structure–Activity Relationship of Lower Chlorinated Biphenyls and Their Human-Relevant Metabolites for Astrocyte Toxicity

**DOI:** 10.1021/acs.chemrestox.3c00095

**Published:** 2023-06-06

**Authors:** Neha Paranjape, Laura E. Dean, Andres Martinez, Ronald B. Tjalkens, Hans-Joachim Lehmler, Jonathan A. Doorn

**Affiliations:** † Department of Pharmaceutical Sciences & Experimental Therapeutics, College of Pharmacy, University of Iowa, Iowa City, Iowa 52242, United States; ‡ Department of Occupational and Environmental Health, College of Public Health, University of Iowa, Iowa City, Iowa 52242, United States; § Department of Civil and Environmental Engineering, IIHR-Hydroscience & Engineering, University of Iowa, Iowa City, Iowa 52242, United States; ∥ Department of Environmental and Radiological Health Sciences, College of Veterinary Medicine and Biomedical Sciences, Colorado State University, Fort Collins, Colorado 80521, United States

## Abstract

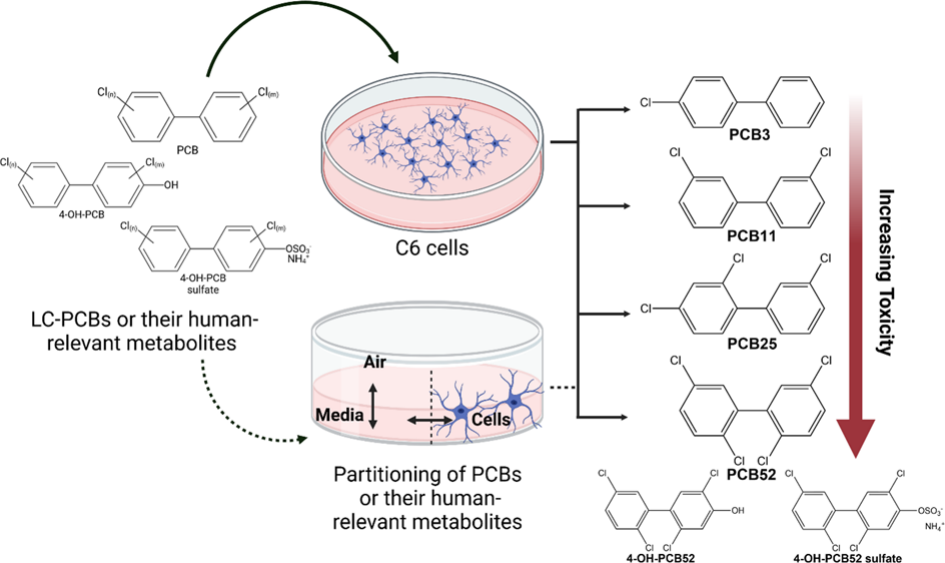

Exposure to polychlorinated biphenyls (PCBs) is associated with developmental neurotoxicity and neurodegenerative disorders; however, the underlying mechanisms of pathogenesis are unknown. Existing literature has focused mainly on using neurons as a model system to study mechanisms of PCB-mediated neurotoxicity, overlooking the role of glial cells, such as astrocytes. As normal brain function is largely astrocyte-dependent, we hypothesize that astrocytes play an important role in PCB-mediated injury to neurons. We assessed the toxicity of two commercial PCB mixtures, Aroclor 1016 and Aroclor 1254, and a non-Aroclor PCB mixture found in residential air called the Cabinet mixture, all of which contain lower chlorinated PCBs (LC-PCBs) found in indoor and outdoor air. We further assessed the toxicity of five abundant airborne LC-PCBs and their corresponding human-relevant metabolites in vitro models of astrocytes, namely, the C6 cell line and primary astrocytes isolated from Sprague–Dawley rats and C57BL/6 mice. PCB52 and its human-relevant hydroxylated and sulfated metabolites were found to be the most toxic compounds. No significant sex-dependent cell viability differences were observed in rat primary astrocytes. Based on the equilibrium partitioning model, it was predicted that the partitioning of LC-PCBs and their corresponding metabolites in biotic and abiotic compartments of the cell culture system is structure-dependent and that the observed toxicity is consistent with this prediction. This study, for the first time, shows that astrocytes are sensitive targets of LC-PCBs and their human-relevant metabolites and that further research to identify mechanistic targets of PCB exposure in glial cells is necessary.

## Introduction

Polychlorinated biphenyls (PCBs) are persistent organochlorine chemicals that were used in many industrial applications before their commercial manufacturing was banned in 1979 by the United States Environmental Protection Agency (U.S. EPA). PCBs constitute 209 structurally similar chlorinated biphenyls, each referred to as a congener.^1^ Despite the ban on commercial manufacturing of PCBs, inadvertent PCB production continues even today, which predominantly comprises lower-chlorinated PCBs (LC-PCBs, PCB congeners containing ≤4 chlorine atom substituents).^1−4^ Due to their volatility as compared to higher-chlorinated PCBs, LC-PCBs have been detected in indoor air samples collected from U.S. schools and outdoor air samples collected from different regions globally.^2,5−7^ PCBs also undergo metabolism in humans, and PCB metabolites have been detected in various human tissue samples;^1,8−10^ however, significant gaps regarding the potential human adverse outcomes of the metabolites remain.

Exposure to PCBs has been associated with neurotoxic outcomes, such as learning and cognitive disabilities, autism spectrum disorders (ASD), and attention deficit hyperactivity disorder.^6,11^ Structurally, PCBs can also be classified as dioxin-like(DL) and non-dioxin-like (NDL), and accumulating evidence suggests that NDL PCBs may be the key players in PCB-induced neurotoxicity.^12^ Although an association between PCB exposure and learning disabilities in children was reported over five decades ago,^13^ the mechanism of PCB-mediated neurotoxic outcomes is not fully understood. Previous in vivo and in vitro studies have used neurons as the model system to study PCB-induced neurotoxicity,^14−16^ greatly advancing this field. However, these studies did not consider the role of glia as targets or mediators of injury following PCB exposure. Glial cells, such as astrocytes, are required to regulate and maintain homeostasis in the central nervous system during health and disease.^17,18^ Astrocytes perform a myriad of trophic and homeostatic functions and are the most abundant type of cells found in the brain.^17,19^ Due to the important role astrocytes play in regulating neuronal function, such as through synapse grooming, providing energy-rich substrates to neurons for ATP synthesis, and maintaining blood–brain barrier integrity,^19,20^ it is imperative to study their involvement in PCB-induced neurotoxicity. However, only a few studies have been published describing the effects of PCBs on astrocytes.^21,22^ Gurley et al. showed that exposure to a commercial mixture of PCBs in C6 cells, Aroclor 1254 (A1254) at 10 ppm, caused increased production of Glial cell-line derived neurotrophic factor and that this effect was abolished when C6 cells were treated with a protein kinase C inhibitor, bisindolymaleimide at 5.6 μM. Another study that was recently published by McCann et al. showed that exposure to A1254 in primary mouse astrocytes caused a dose-dependent increase in oxidative stress. Exposure to 10 μM A1254 caused an increase in the expression of antioxidant response element genes, as well as an increase in glucose uptake. Either 10 or 50 μM A1254 in these cells did not overtly affect the mitochondrial function, as assessed by the Seahorse assay, except that the 10 μM exposure lead to an increase in spare capacity in the primary mouse astrocytes but not at 50 μM.

**Figure 1:**
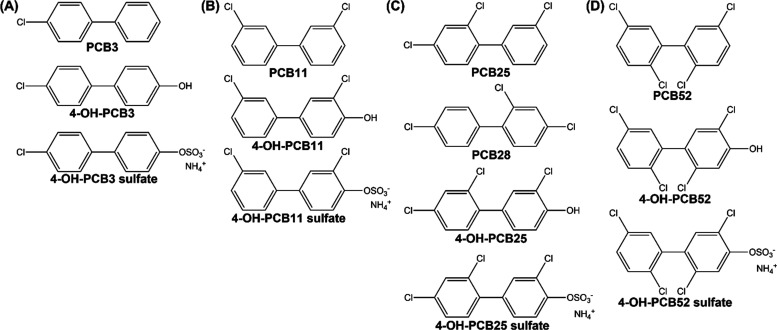
Structure and nomenclature of human-relevant hydroxylated and sulfated metabolites is shown under respective LC-PCB: (A) PCB3 and its metabolites, (B) PCB11 and its metabolites, (C) PCB25, PCB28, and their metabolites, (D) PCB52 and its metabolites.

The current study used cultured astrocytes to analyze the toxicity of Aroclor and non-Aroclor PCB mixtures and five LC-PCBs abundantly found in air, along with their corresponding human-relevant hydroxylated and sulfated metabolites (Figure 1). Two commercial PCB mixtures, Aroclor 1016 (A1016) and A1254, that continue to be released into the environment through capacitors, transformers, and building materials, were included in this study.^2,23^ In addition, the Cabinet mixture, as defined by Herkert et al., was studied as an example of an environmentally relevant, non-Aroclor PCB mixture present in residential air.^24^ LC-PCBs used include 4-chlorobiphenyl (PCB3), 3,3′-dichlorobiphenyl (PCB11), 2,3′,4-trichlorobiphenyl (PCB25), 2,4,4′-trichlorobiphenyl (PCB28), and 2,2′,5,5′-tetrachlorobiphenyl (PCB52) and the corresponding para hydroxylated and sulfated metabolites. Furthermore, the role of sex was considered for rat primary astrocytes. We demonstrate that the toxicity of tested LC-PCBs is structure-dependent. The hydroxylated metabolites are the most toxic compounds compared to sulfated metabolites and the parent LC-PCBs. We also predict the partitioning of LC-PCBs and their human-relevant metabolites in different phases of the abiotic and biotic components of the cell culture system based on the equilibrium partitioning model. The findings of this study predict astrocytes to be a mechanistic target of PCBs with implications in neurotoxic outcomes.

## Experimental Procedures

### Chemicals and Reagents

Aroclor PCB mixtures, A1016 and A1254 (lot number KC 12-638) were provided by the Synthesis Core, Iowa Superfund Research Program (ISRP) from original containers from Monsanto (St. Louis, MO). The PCB congener profiles of both the Aroclor mixtures have been characterized and reported previously.^25,26^ The Cabinet mixture, provided by Synthesis Core, was prepared by mixing 2,2′,4,4′-tetrachlorobiphenyl (PCB 47), 2,2′,4,6′-tetrachlorobiphenyl (PCB 51), and 2,3′,4,5′-tetrachlorobiphenyl (PCB 68) from AccuStandard (New Haven, CT, USA) in a ratio of 75:17:8 by weight.^27,28^ All the LC-PCBs and their corresponding human-relevant hydroxylated and sulfated metabolites used in this study were synthesized and provided by the Synthesis Core, ISRP, as previously described.^29−32^ PCB derivatives were authenticated following published guidelines.^33^

3-(4,5-Dimethylthiazol-2-yl)-2,5-diphenyltetrazolium bromide (MTT) (Cat. No. M5655), cOmplete, Mini Protease Inhibitor cocktail (Cat. No. 11836153001), and the cytotoxicity detection kit (Cat. No. 11644793001) were obtained from Roche (Sigma-Aldrich, St. Louis, MO, U.S.A.). Pierce bicinchoninic acid (BCA) Protein Assay Kit (Cat No. 23227) used for protein estimations was purchased from Thermo Fisher Scientific, (Waltham, MA, U.S.A.). Minimal Essential Medium (MEM) (Cat. No. 11095080), Dulbecco’s Modified Eagle Medium/Nutrient Mixture F-12 (DMEM/F12) (Cat. No. 11320033), Fetal Bovine Serum (FBS) (Cat. No. 16000044), MEM without phenol red (Cat No. 51200038), DMEM/F12 without phenol red (Cat. No. 11039021), Hanks Balanced Salt Solution (HBSS) (Cat. No. 14025092), and Trypsin-EDTA (0.25%) (Cat. No. 25200056) were purchased from Gibco (Thermo Fisher Scientific, Waltham, MA. U.S.A.). Penicillin-Streptomycin (P/S) containing10,000 U/mL (Cat. No. 15140122) was purchased from Life Technologies (Carlsbad, CA, U.S.A.).

### Cell Culture

#### C6 Cells

C6 cells (CCL-107, Lot No. 63821786) were purchased from the American Type Culture Collection (ATCC, Manassas, VA, U.S.A.). This cell line has been previously used as an in vitro astrocyte model for toxicity^22^ and is morphologically and functionally similar to primary astrocyte cultures.^34^ The C6 cells were grown and maintained at 37 °C under humidified conditions at 5% CO_2_ in DMEM/F12 medium supplemented with 10% FBS and 1% P/S. The cells were maintained at a seeding density of 1 × 10^6^ cells in 100 mm tissue culture-treated dishes (Cat. No. 430167, Corning, Somerville, MA, U.S.A.). All the experiments for the current study utilizing C6 cells have been performed within passage 2 and passage 20.

#### Rat Primary Astrocytes

Sprague-Dawley male and female rats were utilized to obtain cultures of sex-specific primary cortical cells. All animal experiments were approved by the Institutional Animal Care and Uses Committee (IACUC) at the University of Iowa. The rat pups were sex-segregated as males and females based on visual examination and later confirmed by the presence of a Y chromosome-specific gene *Sry* by PCR. The gene for beta-actin, *Actb*, was used as a control. The primer sequences used for PCR amplification were as described,^35^ which yielded a 317-bp product for *Sry* and a 220-bp product for *Actb*. Both products were confirmed by gel electrophoresis on a 1% agarose gel after PCR amplification was completed. As previously described, with modifications, astrocytes were obtained from the cortex of PND2/3 rats.^36^ Briefly, cortical sections were dissociated in minimal essential medium with Earle’s salts for suspension cultures (S-MEM; Life Technologies) with 1% penicillin/streptomycin and 1.5 U/mL dispase (Life Technologies) for 60 min, aliquoting and replacing dissociation media every 10 min, adding 8000 U/mL DNase I in water after the first digestion. The enzymatic digestion was stopped by adding a growth medium containing minimal essential medium with Earle’s salts (MEM; Life Technologies) with 1% P/S and 10% heat-inactivated horse serum. After digestion, cells were centrifuged for 10 min at 1000 × *g* at 4 °C, and the pellet was resuspended in a growth medium. Cells were assessed for count and viability and then plated at 10,000 viable cells/cm^2^ in T75 flasks. Flasks were shaken after 24 h, and media was replaced to begin removing microglia and then repeated every 2–3 days. Flow cytometry utilizing GFAP and IBA1 antibodies was run at passage 2 to confirm the elimination of microglia from the primary astrocyte cultures. The rat primary astrocyte cultures were maintained in MEM with 1% P/S and 10% heat-inactivated horse serum at 37 °C under humidified conditions at 5% CO_2_. The detailed protocol for astrocyte isolation of sex segregated rat pups and further validation of purity of astrocyte cultures is published and can be accessed at: dx.doi.org/10.17504/protocols.io.e6nvwjry7lmk/v1.

#### Mouse Primary Glial Cultures

Primary glial cell cultures obtained from PND1-3 C57BL/6 mice pups were provided by Dr. Tjalkens at Colorado State University, Fort Collins, CO, U.S.A. The primary astroglial cell cultures were grown and maintained, as described previously in MEM medium supplemented with 10% FBS and 1% P/S.^37^

#### PCB Exposures

All the LC-PCB exposures or their corresponding human-relevant metabolite exposures were performed in serum-free, phenol-red-free cell culture media suited for the respective cell type described above. The exposures were within a concentration range of 0.5–50 μM for 24 h at 37 °C under humidified conditions at 5% CO_2_. DMSO was used as a negative control. All stock solutions of the test compounds were prepared in DMSO. The final DMSO concentration was maintained at <0.5% for all the exposures. After the 24 h incubation, either an MTT assay for cell viability or an LDH assay for cytotoxicity was employed.

#### MTT Assay

C6 or primary cells were seeded in 24-well cell culture plates (Corning Inc., Corning, NY, U.S.A; Cat No. 3526) and allowed to grow until 80–90% confluent. The cells were then exposed to test compounds, as described above (0, 0.5, 1, 5, 10, 20, and 50 μM). The MTT assay was performed, as described^38^ with modifications. After 24 h of exposure, the media was removed, and cells were washed with HBSS once. Two hundred microliters of 0.5 mg/mL MTT solution prepared in HBSS/Glucose (Glucose used at 1 mg/mL HBSS) was added to each well of the 24-well plate. The cells were incubated with the MTT solution for 45 min to 1 h at 37 °C under humidified conditions at 5% CO_2_. After incubation, the MTT solution was removed, and the water-insoluble purple formazan product was dissolved in 400 μL DMSO. This DMSO solution was added to a clear, flat bottom 96-well polystyrene plate (Cat. No. 12-565-501, Thermo Fisher Scientific, Waltham, MA. U.S.A.) in duplicate and read at 570 and 650 nm using a microplate reader (SpectraMax 190, Molecular Devices, San Jose, CA. U.S.A.). The mean percent cell viability was calculated as follows:
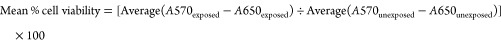


#### LDH Assay

C6 or primary cells were seeded in 96 well cell culture plates (Corning Inc., Corning, NY, U.S.A; Cat No. 3596) and allowed to grow until 80–90% confluent. The cells were then exposed to test compounds, as described above. After 24 h of incubation, lactate dehydrogenase (LDH) released into the culture media, as a measure of cell death, was determined using the Cytotoxicity Detection Kit per the manufacturer’s instructions (Version: 12, Content Version: November 2020).

#### PCB Partitioning Model Prediction

##### Determination of Protein Content in C6 Cells

C6 cells were grown in 100 mm tissue culture treated dishes or T75 cell culture flasks until confluent. Cells were then collected in Radioimmunoprecipitation Assay (RIPA) buffer (Doc. Part No. 2161782, Pub. No. MAN0011565, Rev. B.0, Thermo Fisher Scientific, Waltham, MA. U.S.A.) containing cOmplete, Mini Protease Inhibitor (1 tablet/10 mL RIPA buffer as recommended by the manufacturer). Cells were centrifuged at 14,000 *g* for 15 min. The supernatant was used for protein estimation using BCA assay per the manufacturer’s instructions (Doc. Part No. 2161296, Pub. No. MAN0011430, Rev. B.0, Thermo Fisher Scientific, Waltham, MA. U.S.A.). Pierce BSA Protein Assay Standards (Cat. No. 23208, Thermo Fisher Scientific, Waltham, MA. U.S.A.) were used for generating the standard curves. Protein estimations were conducted in cells from different passages with at least three biological and two technical replicates.

##### Determination of Lipid and Water Content in C6 Cells

C6 cells were grown until approximately 90% confluent, washed twice with DPBS, trypsinized, and cell count was determined. The cells were centrifuged in PBS at 100 × *g* for 10 min, and the PBS was carefully aspirated. Total lipids were extracted as described.^39^ The tubes were pre- and post-weighed to determine lipid content gravimetrically using a microbalance (Mettler-Toledo MT-5). Blank tubes without the C6 cells were extracted in parallel.

C6 cell pellets were obtained as described above for the lipid determinations and freeze-dried (Labconco Freeze dry system Cat. No. 7740020) for 24 h. Cell water content was determined based on pre- and post-lyophilization weights using a microbalance (Mettler-Toledo MT-5).

##### Equilibrium Partitioning Model

Predictions of the individual PCB congener and their metabolite fractions in the cell culture system and inside the cells were carried out using a mass balance approach building on published PCB partitioning models in cell culture systems.^40^ We presumed chemical equilibrium was reached between all involved phases, including the cells, the medium (i.e., freely dissolved), and air in the headspace, and no saturation of the chemicals in the system. Further, we considered three phases inside the cell: storage lipid, liquid/cytosol (i.e., freely dissolved), and protein. Partitioning into the plastic walls was not included due to the slow PCB diffusivity into polystyrene relative to the cells and our short experimental time.^41^ The mass balance for the *i*th chemical can be described as the following equations:^40,42−44^
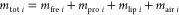
1where *m*_tot *i*_, *m*_fre *i*_, *m*_pro *i*_, *m*_lip *i*_ and *m*_air *i*_ are the masses at equilibrium associated with the total system, freely dissolved, bound to protein and lipid, and partitioned into the air, respectively, for the *i*th chemical (i.e., PCB, OH-PCB and OH-PCB sulfate). Changing masses into concentrations, we can describe eq 1 as follow:
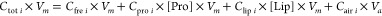
2where *C*_tot *i*_, *C*_fre *i*_, *C*_pro *i*_, *C*_lip *i*_ and *C*_air *i*_ are the concentrations at equilibrium for total, freely dissolved, bound to protein and lipid, and partitioned into air concentrations for the *i*th chemical. [Pro] and [Lip] correspond to the concentrations of protein and lipid in the system, and *V_m_* and *V_a_* are the medium and air headspace volumes. Substituting concentrations of sorbing phases with equilibrium partition coefficients to protein (*K*_pro/*w i*_ = *C*_pro *i*_/*C*_fre *i*_) and lipid (*K*_lip/*w i*_ = *C*_lip *i*_/*C*_fre *i*_), partitioning into air (*K*_air/*w i*_ = *C*_air *i*_/*C*_fre *i*_), dividing by *V_m_* and rearranging eq 2 to describe fractions:
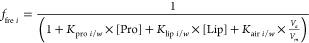
3
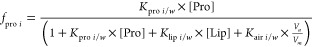
4
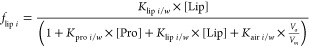
5
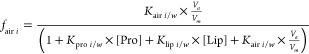
6

We grouped the fractions of protein, lipids, and a fraction of the freely dissolved fraction to estimate the fraction of the chemical corresponding to the cell fraction. The individual equilibrium partition coefficients were calculated from published PP-LFERs via the UFZ-LSER database website.^45^ We noticed that the UFZ-LSER database does not contain OH-PCBs and OH-PCB sulfates (i.e., CAS numbers are not available for OH-PCBs and OH-PCB sulfates), so we used SMILES^46,47^ to generate OH-PCBs and OH-PCB sulfates as inputs. Individual Henry’s law constants for PCBs were obtained from Dunnivant, Elzerman, Jurs, and Hasan^48^ and temperature experiment corrected (37 °C).^49^

### Data Analysis and Statistical Tests

Each experiment was performed at least three times independently, with three analytical replicates in each experiment. Data are represented as mean ± SEM. Multiple tests (nonparametric) were used to compare cell viability between male and female rat primary astrocytes at each concentration. Statistical significance was set to *p* ≤ 0.05. Data were analyzed by GraphPad Prism versions 8.3.0 and above for Windows (GraphPad Software, San Diego, CA, U.S.A.) and R.^50^ The R code that was created to estimate the fraction of PCBs and their metabolites, including Figure 7, is freely available at: https://doi.org/10.5281/zenodo.7909031.^51^

## Results

### Structure–Activity Relationship of PCB Mixtures, LC-PCBs, and Their Human-Relevant Metabolites for Cytotoxicity in C6 Cells

**Figure 2:**
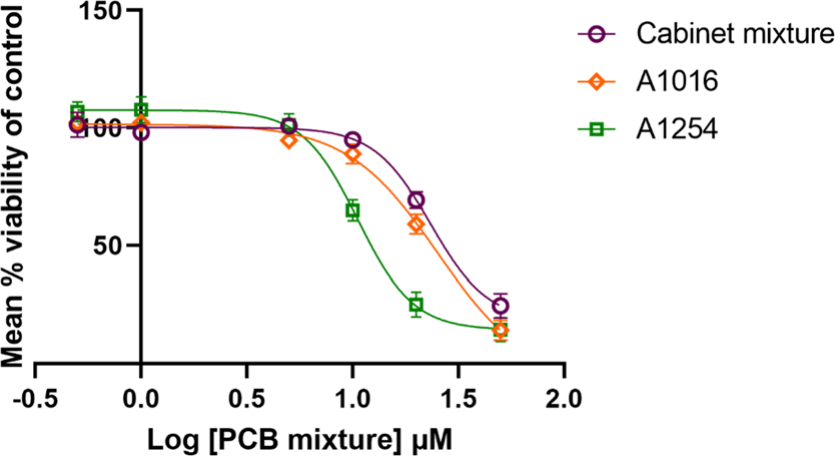
Concentration–response curves of three PCB mixtures analyzed for toxicity in C6 cells after a 24 h exposure. Data is normalized to DMSO control and is based on at least *n* = 3.

We analyzed the toxicity of two commercial PCB mixtures, A1016 and A1254, and a non-Aroclor PCB mixture called the Cabinet mixture. Recent evidence shows that exposure to these three PCB mixtures at physiologically relevant concentrations causes a decrease in cell proliferation and adipogenesis in adipose mesenchymal stem cells.^27^ In the current study, C6 cells were exposed to each mixture separately at a concentration range of 0.5 to 50 μM in phenol red-free and serum-free DMEM/F12 medium. Cell viability was analyzed by MTT assay after 24 h of exposure. The concentration–response curves are represented in Figure 2, and the inhibitory concentration 50 (IC_50_) was found to be 25.2 μM for A1016, 10.6 μM for A1254, and 23.4 μM for the Cabinet mixture (Table 1).

**Table 1:** Comparison of IC_50_ Values of Three PCB Mixtures as Assessed by MTT Assay in C6 Cell Line^a^

PCB mixture	IC_50_ (μM)
Cabinet mixture	23.4
A1016	25.2
A1254	10.6

a Data were obtained from at least three biological replicates with each containing three technical replicates.

**Figure 3:**
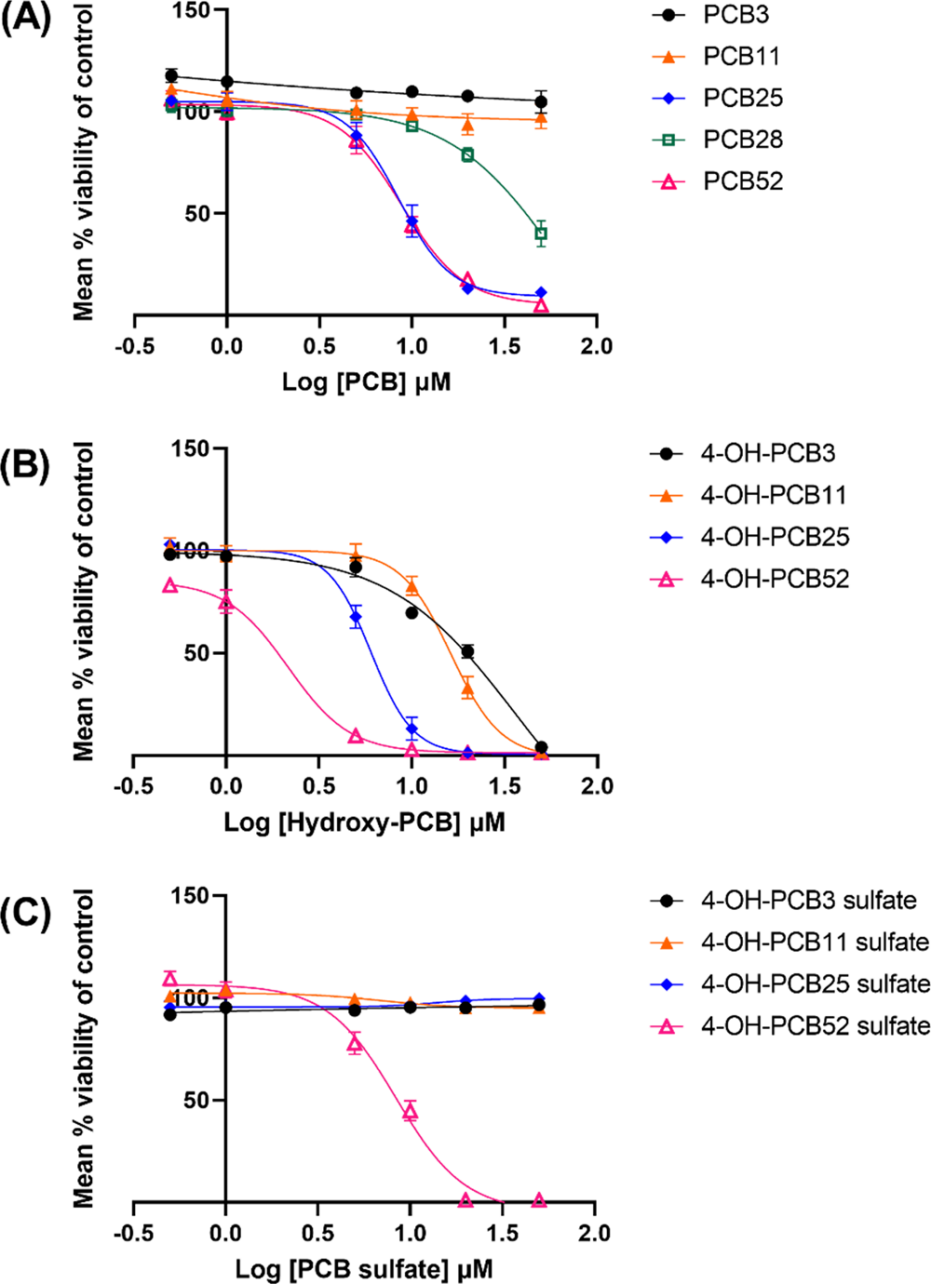
Concentration–response curves of (A) parent LC-PCBs and their (B, C) human-relevant metabolites in C6 cells. A concentration range of 0.5 to 50 μM was used for each compound and cell viability assessed using the MTT assay. Data were obtained from at least three biological replicates with each containing three technical replicates and were normalized to DMSO control. They are represented as mean percent cell viability ± SEM against Log_10_ PCB or PCB-metabolite concentration.

Since the toxicity of PCB congeners depends on the position and number of chlorine atom substitutions on the biphenyl ring,^1^ we further analyzed the toxicity of five abundantly found LC-PCBs in air, along with their human-relevant hydroxylated and sulfated metabolites (Figure 1). C6 cells were exposed for 24 h to these LC-PCBs or their human-relevant metabolites within a concentration range of 0.5 to 50 μM in phenol-red free and serum-free DMEM/F12 medium after which MTT assay was performed to determine cell viability (Figure 3). The IC_50_ was determined where possible (Table 2).

**Table 2:** Comparison of IC_50_ Values of Five Representative LC-PCBs and their Human-Relevant Metabolites as Assessed by MTT Assay in C6 Cell Line^a^

parent compound	no. of chlorine atom substituents	parent compound IC_50_ (μM)	hydroxylated metabolite IC_50_ (μM)	sulfated metabolite IC_50_ (μM)
4-chlorobiphenyl (PCB 3)	1	>50	21.40	>50
3,3′-dichlorobiphenyl (PCB 11)	2	>50	13	>50
2,3′,4-trichlorobiphenyl (PCB 25)	3	10	5.3	>50
2,4,4′-trichlorobiphenyl (PCB28)	3	∼25		
2,2′,5,5′-tetrachlorobiphenyl (PCB 52)	**4**	**8.8**	**2.2**	**8.4**

a Data were obtained from at least three biological replicates with each containing three technical replicates.

Analysis of cell viability using the MTT assay revealed that PCB52 and its metabolites, 4-OH-PCB52 and 4-OH-PCB52 sulfate, were most cytotoxic to C6 cells, with IC_50_ concentrations of 8.8 μM for PCB52, 2.2 μM for 4-OH-PCB52, and 8.4 μM for 4-OH-PCB52 sulfate. The other LC-PCB congeners had IC_50_ values ranging from 10 to >50 μM while the IC_50_ concentrations for hydroxylated and sulfated metabolites ranged from 5.3 to >50 μM. For the parent LC-PCBs, relative cell viability decreased with increasing chlorine atom substitutions on the biphenyl ring. PCB3 and PCB11 were non-toxic in the tested concentration range, while PCB52 was the most toxic LC-PCB congener. PCB25 and PCB28 are both tri-chlorinated PCBs with distinct concentration–response curves, indicating the importance of the position of chlorine atom on the biphenyl ring as an important determinant of toxic outcomes of PCBs. The relative viability of cells exposed to the hydroxylated metabolites also decreased as the number of chlorine atom substitutions on the biphenyl ring increased. Among the sulfated LC-PCB metabolites tested, only 4-OH-PCB52 sulfate reduced cell viability within the tested concentration range.

**Figure 4:**
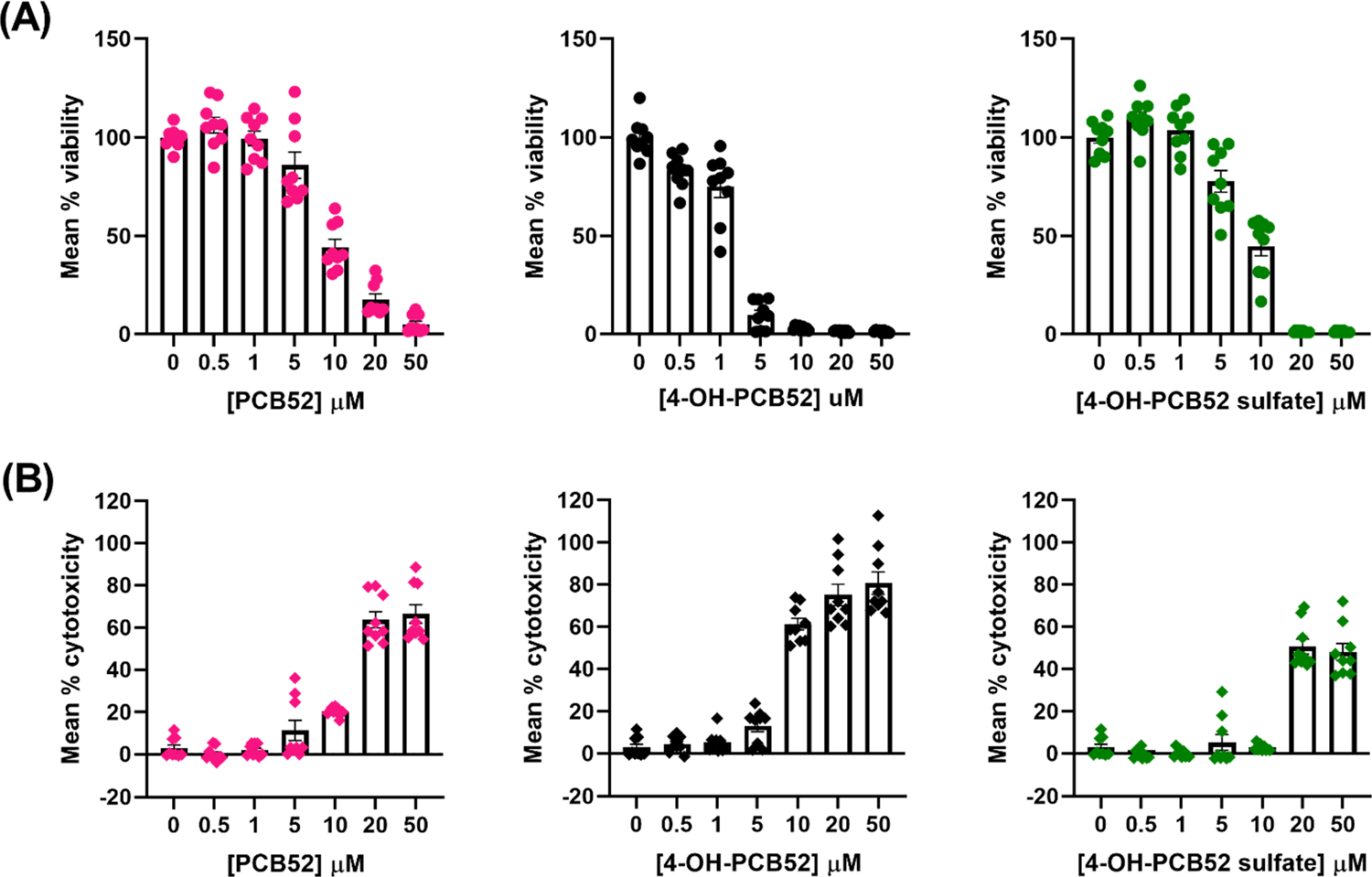
Comparison of toxicity of PCB52 and its human-relevant metabolites as assessed by (A) MTT assay and (B) LDH assay as a measure of cell viability and cell death in C6 cells, resp. Data were obtained from at least three biological replicates with each containing three technical replicates and are represented as mean percent viability ± SEM for MTT assay or mean percent cytotoxicity ± SEM for LDH assay, against the concentration of PCB52 or 4-OH-PCB52 or 4-OH-PCB52 sulfate.

To further characterize the observed toxicity of PCB52 and its human-relevant metabolites in C6 cells, the LDH assay was employed to assess cell death as a function of LDH release from dead cells. The C6 cells were exposed to a similar concentration range as described above. The results indicated similar trends in cytotoxicity as those observed with the MTT assay (Figure 4). The LDH assay relies on the release of LDH from damaged/dead cells and is considered a better measure of cytotoxicity. On the other hand, the MTT assay relies on the metabolic capacity of cells, and hence can be used to estimate cell viability, depending on cellular metabolic capacity. We chose to screen with MTT assay to gain insight into the metabolic capacity of cells over varying concentration ranges, but also incorporated the LDH assay to measure true cytotoxicity of PCB52 and its human-relevant metabolites.

### Cytotoxicity of PCB52 and Its Human-Relevant Metabolites in Primary Astrocytes

**Figure 5:**
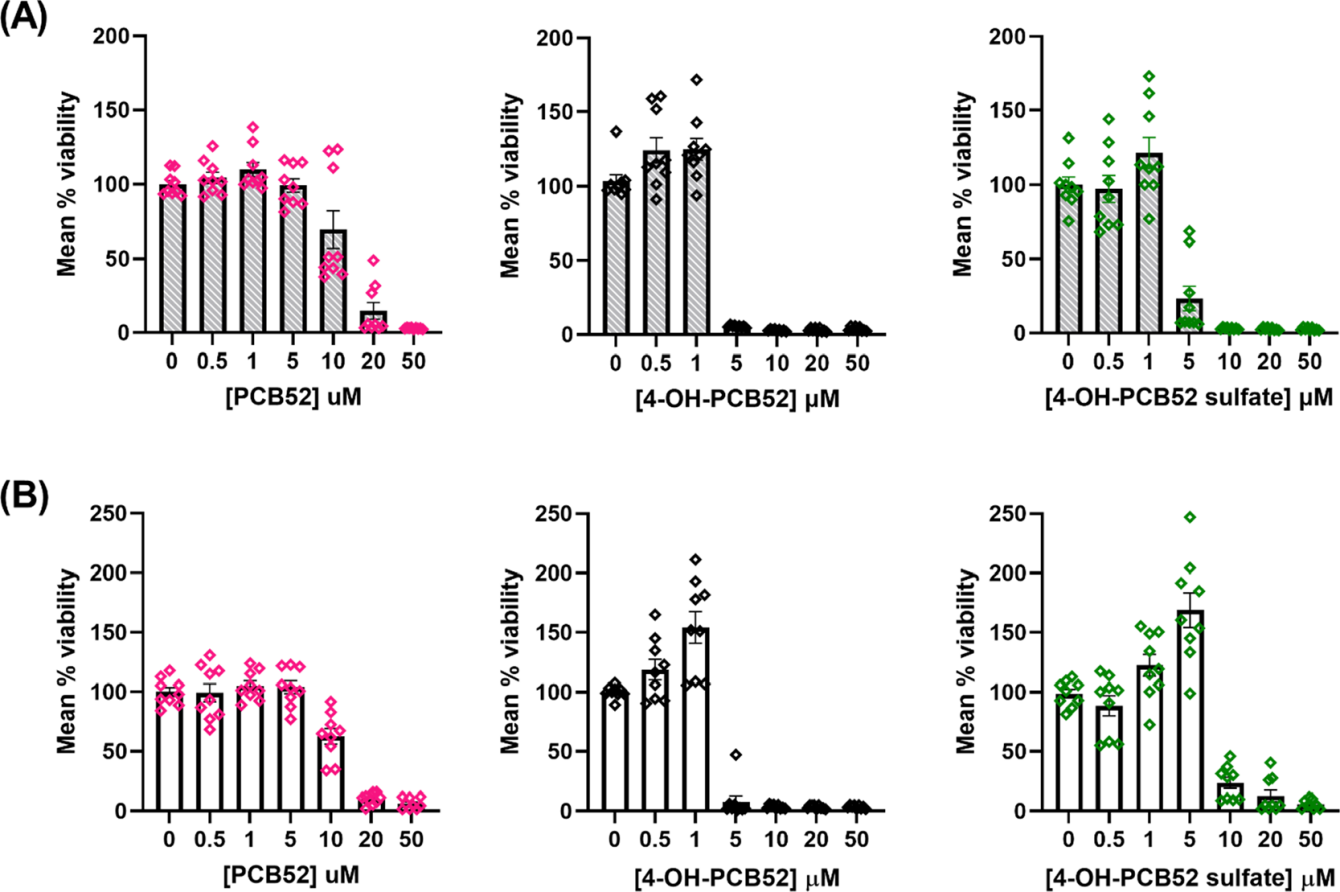
Comparison of toxicity of PCB52 and its human-relevant metabolites as assessed by MTT assay in (A) Primary astrocyte cultures from PND 1-3 Sprague-Dawley rats and (B) primary glial cells from PND 1-3 C57BL/6 mice. The inflection points observed at 1 and 5 μM of 4-OH-PCB52 and 4-OH-PCB52 sulfate respectively, in mouse primary glia are notable and are not as prominently seen in the rat primary astrocytes. Data were obtained from at least three biological replicates with each containing three technical replicates and are represented as mean percent cell viability ± SEM against concentration of PCB52, 4-OH-PCB52, or 4-OH-PCB52 sulfate.

**Figure 6:**
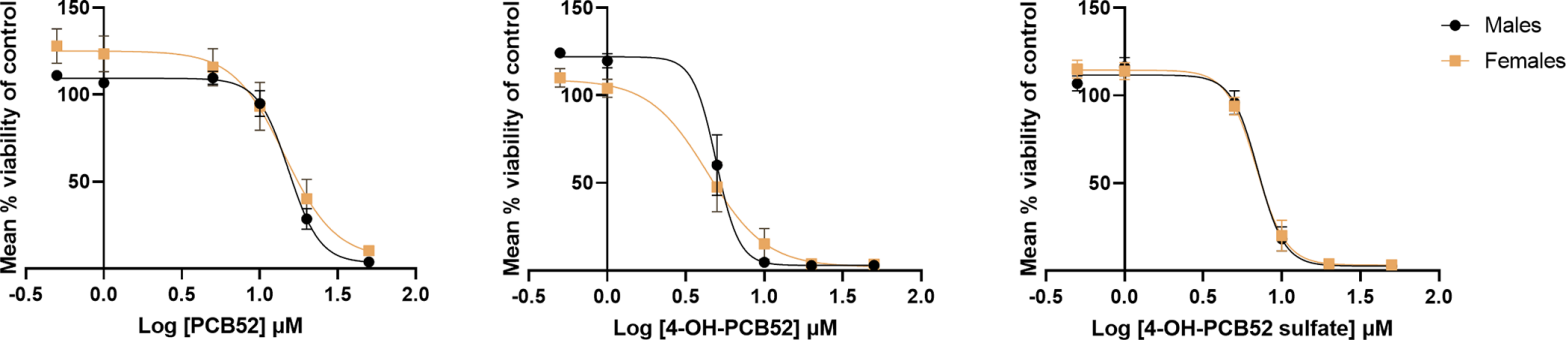
Concentration–response curves of PCB52 or 4-OH-PCB52 or 4-PCB52 sulfate in primary astrocytes obtained from PND 1/2 male or female Sprague-Dawley rat pups. A concentration range of 0.5 to 50 μM was used for each compound and cell viability assessed using the MTT assay. Data were obtained from at least three biological replicates with each containing three technical replicates and were normalized to DMSO control. They are represented as mean percent cell viability ± SEM against Log_10_ PCB or PCB-metabolite concentration.

Due to the abundance of PCB52 in indoor and outdoor air^2,5^ and its toxicity in the C6 cell model, we selected PCB52 and its human-relevant metabolites for further studies in primary astrocyte cultures. Rat primary astrocytes were isolated from postnatal day (PND) 1–3 Sprague-Dawley rat pups and were confirmed to be >95% astrocytes. After the primary astrocytes were 80–90% confluent, they were exposed to 0.5 to 50 μM of PCB52, 4-OH-PCB52, or 4-PCB52 sulfate for 24 h and then analyzed for cell viability by MTT assay (Figure 5A). We also analyzed cell viability in sex-segregated rat astrocytes exposed to PCB52, 4-OH-PCB52, and 4-OH-PCB52 sulfate. The IC_50_ values are shown (Table 3), and no significant sex-dependent differences were seen in the cell viability of PCB52 or its human-relevant metabolites in these cells (Figure 6).

**Table 3:** Comparison of IC_50_ Values of PCB52 and its Human-Relevant Metabolites as Assessed by MTT Assay in Sex Segregated Rat Primary Astrocytes^a^

PCB mixture	IC_50_ males (μM)	IC_50_ females (μM)
PCB52	15.3	14.4
4-OH-PCB52	4.9	4.4
4-OH-PCB52 sulfate	7.0	6.9

a Data were obtained from at least three biological replicates.

The toxicity of PCB52 and its human-relevant metabolites was also assessed in primary glial cells isolated from PND 1-3 C57BL/6 mouse pups (Figure 5B) and were compared with the results from similar analysis in mixed primary astrocyte cultures from rats (Figure 5A). It should be noted that the primary glial cell cultures from mice were about 80% astrocytes and 20% microglia, as opposed to >95% pure astrocyte cultures obtained from rats. Due to the shape of the concentration–response curve, the IC_50_ values could not be calculated for all the compounds (Figure 5). However, an IC_50_ of 11.8 μM in rat primary astrocytes and 9.8 μM in mouse primary mixed glial cells was determined for PCB52. An inflection point, i.e., a significant increase in cell viability at lower concentrations of the test compound, followed by a drastic reduction in cell viability at the next higher concentration, was observed in primary glial cells from C57BL/6 mice (Figure 5). For example, the cell viability increased to 150% at 1 μM of 4-OH-PCB52 but decreased to nearly 0% at 5 μM of 4-OH-PCB52. Similarly, 5 μM of 4-OH-PCB52 sulfate had a significantly higher viability of over 150%, whereas the next higher concentration showed a significantly decreased viability of 20%. Such an inflection point was not as conspicuous in rat primary astrocyte cultures; however, a similar trend of increased cell viability at lower concentrations followed by a drastic reduction in cell viability at the next highest concentration was observed.

### Equilibrium Partitioning Model of LC-PCBs and Their Human-Relevant Metabolites In Vitro

The partitioning of LC-PCBs and their human-relevant metabolites in a cell culture system depends on their lipophilicity and volatility and the composition of the different compartments of the cell culture model. Because the extent to which PCBs or their human-relevant metabolites can enter or bind to the cells determines their toxicity, we estimated the partitioning of PCBs and their metabolites within different biotic and abiotic compartments of the cell culture system using a mass balance approach.^40,42−44^ These estimates are based on the physicochemical properties that are experimentally determined for PCBs and calculated for human-relevant PCB metabolites and the protein, lipid, and water composition of the cell culture system. Because the latter information is not available for C6 cells, we determined the protein, lipid, and water content of C6 cells experimentally. The protein, lipid, and water content of C6 cells were 1.18 × 10^–4^ ± 8.25 × 10^–6^, 9.57 × 10^–5^ ± 9.21 × 10^–6^, and 2.84 × 10^–6^ ± 1.18 × 10^–7^ μL/cell, respectively.

**Figure 7:**
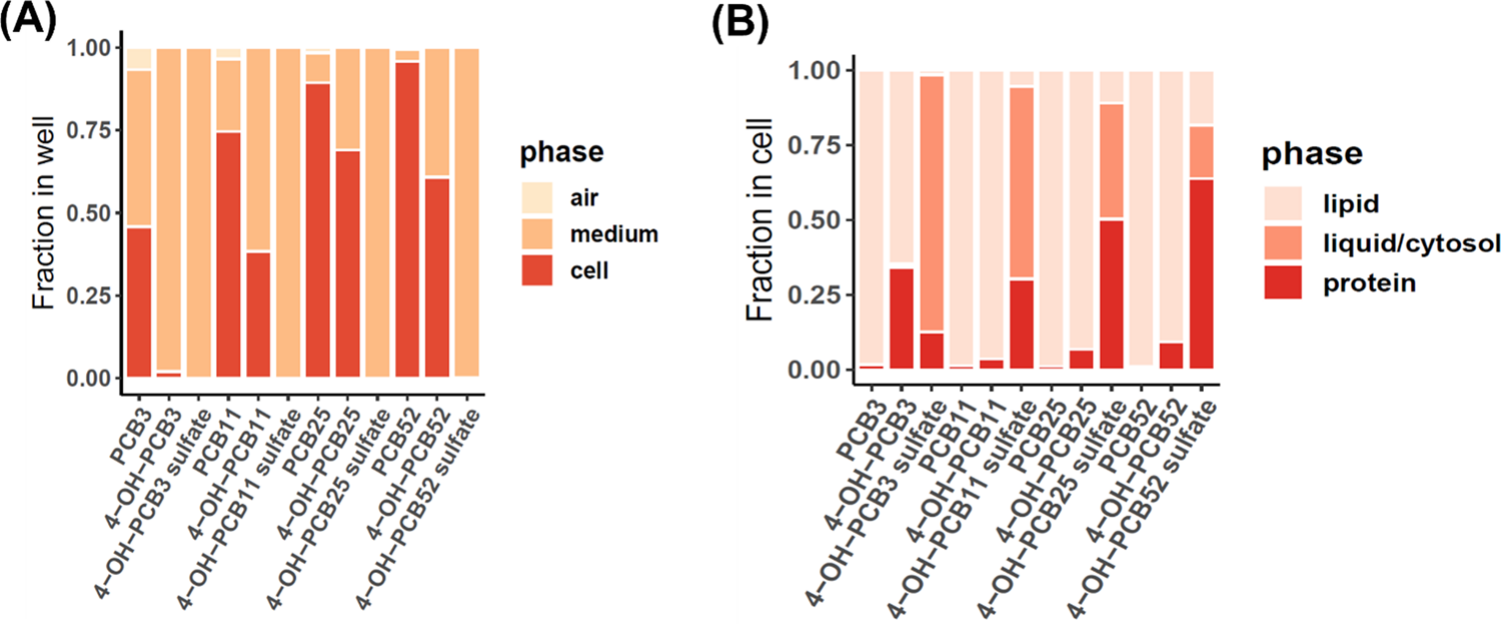
Prediction of partitioning of tested LC-PCBs and their human-relevant hydroxylated and sulfated metabolites in different phases based on the equilibrium partitioning model. Fraction of PCBs partitioning in each phase of the (A) cell culture system and (B) the C6 cells has been shown.

The partitioning of the different LC-PCB metabolites reveals significant differences in how these compounds can partition into the cells (Figure 7). The partitioning of the parent LC-PCBs increases with increasing chlorination. PCB52 is predicted to partition preferentially into the lipid-rich ‘cell’ phase. Similarly, the OH-PCBs partition between the ‘cell’ and ‘medium’ phases in the well, with the fraction of the OH-PCB associated with the cells increasing with increasing chlorination. In contrast, the sulfated metabolites are hydrophilic because of the negatively charged sulfate group and are predicted to remain almost entirely in the ‘medium’ phase. The equilibrium partitioning model also suggests that a small fraction of the parent LC-PCBs may be released into the air over the 24 h incubation period, especially for the more volatile PCB3.

Within the C6 cells, the model predicts that the parent LC-PCB congeners largely partition to the lipid phase, whereas OH-PCBs partition between the lipid and protein phases. The sulfated metabolites partition between all three phases to varying degrees. As chlorination increases, the lipophilicity of PCB sulfates increases; hence, a larger fraction of those metabolites partition between the lipid and protein phases of the cell.

## Discussion

The first evidence linking exposure to PCBs and the development of cognitive dysfunction appeared in 1968 when accidental exposure to PCBs occurred through the consumption of contaminated rice bran oil.^13^ Subsequent epidemiological and preclinical studies consistently suggest that PCB exposure impairs cognitive function and memory and may increase the risk of ASD outcomes and intellectual disability.^6,52−54^ In addition, PCB exposure has recently been implicated in neurodegenerative diseases, such as Parkinson’s disease.^14^ Findings from an assessment of postmortem human brain samples from neonatal and adult brains show that higher chlorinated PCB detection frequencies and levels are higher in adult brains than in neonatal brains.^10^ This observation further implies that LC-PCBs may be important players in developmental neurotoxicity, whereas the higher chlorinated PCBs may play a role in neurodegeneration. The same study also identified hydroxylated PCB metabolites in human brain samples. These studies further warrant the assessment of the toxicity of individual LC-PCB congeners and their human-relevant hydroxylated and sulfated metabolites.

We know about the mechanisms of PCB-induced neurotoxicity largely from models assessing PCB-induced toxic outcomes in cultured neurons or neuronal cell lines. In rat cortical neurons, PCB11 increased dendritic arborization through a mechanism involving cAMP response element binding protein.^55^ Animal and in vitro studies show that exposure to PCBs causes a reduction in dopamine content in the rat brain and dopaminergic cell culture systems, such as PC12 cells.^14,56,57^ In addition, PCBs have been shown to activate the ryanodine receptors (RyR), which regulate calcium flux out of the endoplasmic reticulum,^11^ implicated in neurotoxic outcomes associated with PCB exposure. Mechanisms, such as the activity of PCBs toward RyR and disruption of dopaminergic pathways have been used to establish neurotoxic equivalency schemes to predict the exposure concentrations of PCBs that may be neurotoxic.^58^

Increasing evidence shows that astrocytes serve critical functions required in maintaining health and homeostasis in the brain and protecting it from xenobiotic exposures.^17,19−21^ For example, a recent study used primary rat astrocyte cultures to assess the toxicity of A1254, a commercial PCB mixture. This study showed that A1254 increased oxidative stress, ATP production, and spare capacity of mitochondria, and an elevated expression of antioxidant genes.^59^ However, this study did not investigate individual congeners and their metabolites, particularly LC-PCBs found in indoor air.^2,7,24^

The current study is the first to analyze the toxicity of LC-PCBs as documented mixtures and individual congeners, along with their respective hydroxylated and sulfated metabolites, in cell culture models of astrocytes. Our results indicate that the toxicity of LC-PCBs and their human-relevant metabolites in C6 cells is PCB congener dependent. Our analysis of individual PCB congeners and PCB mixtures suggests that only a few LC-PCBs from a PCB mixture may drive the toxicity caused by that PCB mixture. Thus, in the future, it would be important to assess the PCB congener profiles from environmental samples to better predict the toxicity of PCB mixtures. The metabolites and parent LC-PCBs show distinct concentration–response curves indicating different mechanisms and rate of uptake and/or metabolism in C6 cells and the primary astrocytes resulting in observed toxicity. The findings from concentration–response analysis of LC-PCBs and their human-relevant metabolites are congruent in rat primary astrocytes and C6 cells. Our data also suggest that astrocytes are more sensitive to the LC-PCBs and their human-relevant metabolites than neuronal cells, as seen upon comparing the IC_50_ values from similarly conducted studies.^15^ Rodriguez et al. also show that toxicity of PCBs and their metabolites, as assessed by MTT cell viability assays, is dependent on the rate of uptake and metabolism of a particular cell type. For instance, in the same study, it was shown that HepG2 cells (a human hepatoma cell line) are able to transform 4-OH-PCB3 to 4-OH-PCB3 sulfate and vice versa, but not the SH-SY5Y (human neuronal cell line) or the N27 (rat neuronal cell line) cells, when analyzed after 24 h exposure. Due to the dynamic yet tightly controlled interactions between astrocytes and neurons necessary for normal neuronal function,^17^ a mechanistic understanding of PCB-induced neurotoxicity would be incomplete without taking into account these interactions in future studies.

The inflection points observed in concentration–response curves of 4-OH-PCB52 and 4-OH-PCB52 sulfate in mouse primary glial cultures (∼80% astrocytes; ∼20% microglia) are not as evident in the primary rat astrocyte cultures (>95% astrocytes; <5% microglia) (Figure 6). The varying proportion of microglia in primary astrocyte cultures may be responsible for the observed inflection in cell viability, as microglia can undergo proliferation when activated by chemical exposures, which may further lead to the activation and proliferation of astrocytes.^60^ This finding may thus be indicative of the role of microglia or astrocyte–microglia interactions. Under stressed conditions, such as physical injury, exposure to xenobiotics, or endogenous chemical insults, astrocytes can assume either neuroprotective or neuro-damaging functions and hence can bring about pathological changes in the brain via continued crosstalk and coordination with neurons and microglia.^17,60,61^ Therefore, future study needs to address glial cell interactions and neuron–glia crosstalk in elucidating the mechanisms of neurotoxicity.

The current study also uses sex-segregated rat astrocytes and reports no significant sex-dependent differences in the effect of PCB52 or its human-relevant metabolites on cell viability. However, it must be noted that these findings may not necessarily extend to other functional assays in primary astrocytes, such as those looking at mitochondrial function or calcium uptake and release. The sex differences may also become more apparent if the astrocytes are isolated from older rat pups because of the differences in astrocyte development in males and females with age. According to a recent study, astrocytes show distinct ‘early’ and ‘late’ phenotypes during mouse development.^62^ The ‘late’ phenotype emerges by PND7 in males while it emerges by PND14 in females.^62^ An analogous study is currently unavailable for rat models. However, such factors are important when studying sex-dependent differences in primary astrocyte cultures. In addition, certain PCBs, including PCB52, and some PCB-sulfates have been shown to activate estrogen receptors or have estrogenic effects, which will need to be considered when performing functional assays to study the effects of PCBs on astrocytes and related cells.^63,64^

Even though the absolute values of IC_50_ concentrations of PCB52 and its metabolites in C6 cells (Table 2) and sex-segregated primary astrocytes (Table 3) are different, the trends in toxicity are consistent and comparable. The 4-OH-PCB52 sulfate seems to be more toxic in primary astrocytes as compared to the C6 cell line.

The prediction of PCB partitioning based on the equilibrium partitioning model suggests that the parent LC-PCB congeners are predominantly lipophilic. However, their toxicity varies depending on their chlorination pattern, which may govern the cellular interactions of these LC-PCBs with target molecules. The hydroxylated metabolites likely enter cells and can have various cellular targets for interaction resulting in observed high toxicity. On the other hand, sulfated metabolites are more hydrophilic and remain in the medium per the model prediction. The findings from cell viability assays are consistent with this prediction for sulfated metabolites of PCB3, PCB11, and PCB25. On the other hand, the toxicity of 4-OH-PCB52 sulfate is not consistent with the model prediction. For example, 4-OH-PCB52 sulfate was found to be similarly toxic as PCB52 in C6 cells and even more toxic than PCB52 in rat primary astrocytes. This finding could imply that the PCB52 sulfate may enter the cells, unlike sulfated metabolites of other LC-PCBs. As shown in an earlier study, 4-OH-PCB52 sulfate may be taken up by the cells and undergo hydrolysis to form 4-OH-PCB52, resulting in the observed toxicity.^15^ The chlorination pattern and the presence of ortho-substituted chlorines in PCB52 may also contribute to the observed toxicity of 4-OH-PCB52 sulfate.^15^

Our study shows that LC-PCBs and their human-relevant metabolites elicit structure-dependent responses as assessed by cell viability and cytotoxicity assays. Our findings implicate astrocytes as an overlooked cellular target of PCBs. In addition, these data suggest that LC-PCBs and human-relevant metabolites have distinct molecular targets conferring sensitivity in astrocytes which may determine the adverse outcome pathways associated with each of those compounds. Further study is needed to elucidate the mechanistic targets for parent LC-PCBs and their human-relevant metabolites in astrocytes.

## Abbreviations

ADHD: attention deficit hyperactivity disorder
ASD: autism spectrum disorder
ATCC: American Type Culture Collection
BBB: blood brain barrier
BCA: bicinchoninic acid
CREB: cAMP response element binding protein
DL: dioxin like
DMEM/F12: Dulbecco’s Modified Eagle Medium/Nutrient Mixture F-12
FBS: fetal bovine serum
HBSS: Hanks balanced salt solution
IC_50_: inhibitory concentration 50
ISRP: Iowa Superfund Research Program
IUCAC: Institutional Animal Care and Uses Committee
LC-PCBs: lower-chlorinated biphenyls
LDH: lactate dehydrogenase
MEM: minimal essential medium
MTT: 3-(4,5-Dimethylthiazol-2-yl)-2,5-diphenyltetrazolium bromide
NDL: non-dioxin like
P/S: penicillin–streptomycin
PCBs: polychlorinated biphenyls
PND: postnatal day
RIPA: radioimmunoprecipitation assay
RyR: ryanodine receptors
S-MEM: minimal essential medium with Earle’s salts for suspension cultures
U.S. EPA: United States Environmental Protection Agency
